# The impact of a maternal mental health intervention on intimate partner violence in Northern Ghana and the mediating roles of social support and couple communication: secondary analysis of a cluster randomized controlled trial

**DOI:** 10.1186/s12889-021-12121-9

**Published:** 2021-11-04

**Authors:** Jiepin Cao, John A. Gallis, Mohammed Ali, Margaret Lillie, Safiyatu Abubakr-Bibilazu, Haliq Adam, Elena McEwan, John Koku Awoonor-Williams, John Hembling, Joy Noel Baumgartner

**Affiliations:** 1 School of Nursing, Duke University, 307 Trent Drive, Durham, NC 27705 USA; 2 Duke Global Health Institute, Duke University, Durham, NC USA; 3 Department of Biostatistics and Bioinformatics, Duke University, Durham, NC USA; 4 Catholic Relief Services Country Office, Tamale, Ghana; 5 Catholic Relief Services Head Quarters, Baltimore, MD USA; 6 Ghana Health Service, Accra, Ghana; 7 School of Social Work, The University of North Carolina at Chapel Hill, Chapel Hill, NC USA

**Keywords:** Domestic violence, Gender-based violence, Women, Low- and middle-income countries, Africa south of the Sahara, Intervention, Mediation

## Abstract

**Backgrounds:**

Diverse intervention efforts are implemented to address intimate partner violence (IPV) against women. Via a syndemics theory lens and emerging empirical evidence, mental health interventions demonstrate promise to partially ameliorate IPV. However, the mechanisms of change underlying many IPV interventions are not well understood. These gaps impede our efforts to strengthen or integrate effective components into the current mental health resources, especially in low- and middle-income countries (LMICs). This study aims to examine the impact of a maternal mental health intervention called *Integrated Mothers and Babies Course & Early Childhood Development (iMBC/ECD)* on IPV and whether social support and/or couple communication mediates the intervention effects among women in rural, Northern Ghana.

**Methods:**

The current study is a secondary data analysis of a cluster randomized controlled trial. IPV was measured at baseline and 8 months post-intervention (~ 19 months post-baseline). At baseline, 84.8% of the women enrolled in the study (*n* = 374) reported some type of IPV in the past 12 months. Logistic regression models and multiple mediation analyses were used to address the study aims.

**Results:**

*iMBC/ECD* did not reduce IPV in the intervention group compared to the control group. Social support and couple communication did not mediate the intervention effects on IPV as indicated by the indirect effects of the multiple mediation models. However, increase in social support reduced women’s odds of experiencing emotional violence by 7%, odds ratio (OR) = 0.93, *p* = 0.007; b = − 0.07, 95% confidence interval (CI) = (− 0.13, − 0.02), and improvement in couple communication demonstrated promise in reducing women’s odds of experiencing controlling behaviors by 7%, OR = 0.93, *p* = 0.07; b = − 0.07, CI = (− 0.14, 0.005), though the improvements were not due to the intervention.

**Conclusion:**

This maternal mental health intervention did not reduce IPV; however, the findings extend our knowledge about the impact of such interventions on IPV and the potential mechanisms of change via social support and couple communication. Future research evaluating the impact of mental health interventions on IPV and mechanisms of change is essential for the development of effective interventions. Future programs addressing IPV in LMICs should consider risk factors beyond relationship level (e.g. poverty and gender inequity).

**Trial registration:**

ClinicalTrials.gov # NCT03665246, Registered on August 20th, 2018.

**Supplementary Information:**

The online version contains supplementary material available at 10.1186/s12889-021-12121-9.

## Background

Intimate partner violence (IPV) is defined as violent behaviors, including physical, emotional, and sexual violence as well as controlling behaviors from current or past intimate partners [1]. IPV is a global health challenge and a violation of human rights. Worldwide, women bear the heaviest burden of IPV, with almost one third of women of reproductive age having experienced IPV [2]. The detrimental outcomes of IPV among women cover a variety of life spheres, including physical and sexual health such as traumatic brain injury and HIV infection [3, 4], mental health such as depression and post-traumatic stress disorder [5]; and health of their children across mental, behavioral, and social domains [6, 7]. The harmful impacts of IPV are amplified during pregnancy and postpartum, a potentially stressful period with unique challenges for women of reproductive age [8]. For example, perinatal deaths are 3 times more likely to happen to women who experience IPV during pregnancy compared to those who do not [9].

IPV tends to be even more prevalent among women in low- and middle-income countries (LMICs), with global prevalence estimates ranging from 24.6 to 37.0% [10]. In Ghana, 38.7% of ever-married women of reproductive age have experienced physical, emotional, or sexual violence by their partners during their lifetime [11]. The intersection of patriarchy and poverty consistently adds to women’s vulnerability to IPV, especially in Northern Ghana [12]. Poverty, though prevalent in Ghana, has a gendered negative impact on women wherein women experience more severe poverty compared to men [13], leaving them dependent upon men for resources and vulnerable to IPV.

### The Syndemic of IPV and depression

The relationship between IPV and depression among Ghanaian women can be best described and conceptualized with the syndemics theory, which highlights the co-occurring and mutually exacerbating nature of IPV and depression driven by an adverse social environment. Syndemics, first introduced by Singer (2000), describes the clustering of two or more epidemics (e.g. IPV, depression, substance use, and HIV) that are interrelated and mutually reinforcing and whose synergistic effects consistently deteriorate the health of vulnerable populations [14]. Furthermore, syndemics should be understood as the consequences of disadvantaged social conditions or relationships (e.g. poverty and lack of access to health care services) [15]. Specifically, gender inequity and disadvantaged socioeconomic status are conceptualized to be shared drivers of IPV and depression among women in LMICs [16]. Empirically, there is growing evidence of the syndemic of IPV and depression. Women who experience IPV during pregnancy are 1.69–3.76 and 1.46–7.04 times more likely to report ante- and postnatal depression in LMICs compared to those without IPV exposure, according to a meta-analysis [16]. Similarly, evidence from LMICs including Ghana further reveales that depressive symptoms also expose women to higher risk for IPV compared to those without depressive symptoms [17, 18]. The syndemic of IPV and depression further deteriorates the health of women. For example, the co-occurrence of IPV and depression have synergically contributed to a greater risk of negative health outcomes, including HIV and sexually transmitted infections, for women in Uganda [19]. Though not empirically examined as shared drivers of the IPV and depression syndemic, poverty and gender inequality are corroborated by evidence as drivers for IPV [20] and mental health [21] respectively among Ghanaian women. The syndemics theory highlights the potential for an intervention targeting one epidemic within a syndemic to address another epidemic.

### Could mental health interventions reduce IPV: what does the current evidence say?

Emerging evidence indicates that mental health interventions can also be effective strategies to reduce IPV beyond the promise implied by the syndemics theory. A systematic review examining the effects of mental health or substance abuse treatment on IPV prevention and reduction in LMICs identifies and includes two interventions focused on depression, both of which significantly reduced IPV in the short term [22]. However, none of the studies examined the mechanisms of change underlying the IPV reduction [23, 24]. Thus, the authors suggest that future studies should further assess whether interventions focused on depression can reduce IPV and also identify mechanisms of how mental health interventions impacts IPV [22].

### Social support and couple communication: potential mechanisms of change for IPV programs?

Social support, defined as the perception or evaluation of the functional resources in one’s interpersonal relationships (e.g. emotional support, such as love and care, and instrumental/tangible support that is physically available) [25], has generally been a consistent and stable factor protecting women from experiencing IPV across different populations [26]. With the call to effectively engage members in women’s informal social networks, such as family members, friends, colleagues, and neighbors, with formal services to support women experiencing IPV [27], strengthening social support has been a commonly recommended component in many current programs to prevent and/or respond to IPV [28, 29]. SASA!, a community mobilization intervention conducted in Uganda, is an exemplary intervention to prevent IPV. This program addressed multiple sociocultural factors for IPV, including social support, by applying strategies such as training community activists to engage stakeholders and other community members and to strengthen community connections to change IPV-relevant attitudes and norms [30]. However, the current evidence on the effects of social support-focused interventions on IPV is not conclusive. In a recent systematic review of such interventions, Ogbe and colleagues [31] found that most of the 27 studies (including interventions focused on individual women and interventions focused on a hybrid of individual, network, and community) resulted in positive change in social support post-intervention, though their effects on reducing IPV were not consistently well-supported. Surprisingly, the relationships between the change in social support and IPV outcomes were rarely empirically tested among studies showing a reduction in IPV. This leaves the potential mechanisms of change, such as the mediating role of social support, unexamined and future studies are warranted to assess and confirm how IPV was reduced.

Couple communication is an essential component of intimate relationship functionality and is frequently integrated into healthy intimate relationship programs for IPV prevention and intervention. The close associations between couple communication and IPV are well established [32, 33], wherein poor communication can lead to severe relationship conflicts and later escalate into IPV [34], and difficulty in couple communication is a leading motivation for perpetrators of IPV [35]. According to the Centers for Disease Control and Prevention, programs using strategies to build skills for healthy relationships, including or focused on couple communication skills (e.g. Premarital Relationship Enhancement Program (PREP) [36] or ePREP [37]), have some of the best evidence for helping prevent IPV victimization in the U.S. population [28]. However, the existing programs successfully reducing IPV in Sub-Saharan Africa cannot explain the underlying mechanisms [20]. For example, Microfinance for AIDS and Gender Equity (IMAGE) led to an improvement in couple communication and a reduction in IPV, though the role of couple communication was not empirically tested [38]. In addition, the majority of these programs are primarily designed to address HIV, with the communication component usually specific to HIV risks. Minnis and colleagues acknowledged the importance of future program efforts on general communication skills as the initial step prior to HIV risk communication after their program in South Africa failed to improve relationship communication on HIV [39]. Thus, future research is warranted to test mechanisms of IPV reduction in programs that focus on general couple communication skills.

### Current study

This study contributes to the current literature on the impact of mental health interventions on IPV and the mechanisms of change among rural women in the West Mamprusi and Nabdam Districts of Ghana. Based on the syndemics theory and evidence reviewed above, the first aim of the current study is to evaluate the impact of a group-based maternal mental health intervention called *Integrated Mothers and Babies Course & Early Childhood Development* (iMBC/ECD) on experiences of IPV among rural women in Ghana compared to women in the control group who only received routine group-based health education. All women in the study were part of groups called Community Pregnancy Surveillance and Targeted Education Sessions (C-PrES). The second aim is to examine the mediation effects of social support and couple communication. Specifically, two sets of hypotheses proposed for any IPV and for each subtype of IPV ~ 8 months post-intervention respectively:

- **Hypothesis 1** Women in the intervention group (iMBC/ECD + C-PrES) would have a significantly lower 12-month IPV prevalence compared to those in the control group (C-PrES only).
- **Hypothesis 2** Social support and/or couple communication would mediate the effects of the intervention on IPV.
- **Hypothesis 2a** Social support would mediate the effects of the intervention on IPV.
- **Hypothesis 2b** Couple communication would mediate the effects of the intervention on IPV.

## Methods

### Study design

The current study is a secondary data analysis of a cluster randomized controlled trial (cRCT) designed to evaluate the impact of the iMBC/ECD program delivered by Catholic Relief Services (CRS) on the mental well-being of mothers with children under 2 years of age and the age-appropriate development of their children (ClinicalTrials.gov # NCT03665246, registered on August 20th, 2018). The intervention started in December 2018 and ended in July 2019. Data were collected at baseline (August 2018), pre-intervention/mini survey (December 2018), immediate post-intervention (July 2019), and ~ 8 months post-intervention (February 2020). Due to a delay of 3 months in starting the program, a pre-intervention mini survey was conducted to ensure no significant changes in the primary outcome of interest (depression, measured by PHQ-9) between baseline and pre-intervention. For the current study, the data collected at baseline, immediate post-intervention and ~ 8 months post-intervention were used.

### Recruitment, settings, and participants

A total of 32 communities were recruited, located in the West Mamprusi Municipality (North East Region; Mampruli is spoken) and Nabdam District (Upper East Region; Nabt is spoken) in North Ghana. Eligible participants enrolled in the group-based C-PrES platform administered by CRS were at least 16 years in age, pregnant at baseline, maintained residence within the community during the program (at least 6 months), and were willing to be followed for up to 24 months. C-PrES groups were randomly assigned 1:1 to the intervention group (iMBC/ECD+ C-PrES) or control group (C-PrES only). The cRCT was not blinded to participants, data collectors, and analysis team. More detailed information on sample size, randomization, and blinding is published elsewhere [40].

### Intervention

The iMBC/ECD intervention was designed to lower women’s risk for depression and improve ECD; it was not specifically intended to reduce IPV [41]. As an evidence-based intervention developed in the U.S. [42] driven by cognitive-behavioral therapy strategies and attachment theory principles, the iMBC content supports women to manage their realities (feelings, thoughts, behaviors) in a healthy way. Integrated with the iMBC and messages of ECD, the mechanisms are intended to improve women’s knowledge and skills on mood management, ECD-promoting behaviors, and coping skills; and to enhance women’s perceived social support. The topics discussed in the iMBC/ECD sessions cover effective management of everyday stressors and resilience development (e.g. stressor identification, relaxation, communication skills, and identification of people who offered support) and knowledge and skills on ECD.

The control groups attended routine C-PrES, which promoted maternal, newborn, child health; and nutrition practices such as timely antenatal/postnatal care, institutional deliveries, optimal nutrition/feeding, and management of childhood illnesses. CRS, working in collaboration with the Ghana Health Service (GHS) and their community health workers, identified all pregnant women in the study communities for potential participation and enrollment in the groups, both iMBC/ECD + C-PrES and C-PrES only.

The iMBC/ECD groups had 14 1-h group sessions in total. The groups met every 2 weeks for 7 months. C-PrES only groups were delivered at the same frequency. The iMBC/ECD program also included monthly home visits as needed over the 7-month intervention period to check on the mothers’ mood, assess uptake of negotiated ECD behaviors, and encourage husbands and grandmothers to support mothers’ participation in the project. Five iMBC booster sessions were provided when the iMBC/ECD ended after the 14 sessions over a 10-month period for women in the intervention group. iMBC/ECD was implemented by lay counselors, who were women from local communities, under the supervision of GHS community health officers and CRS field staff. More detailed information on the intervention and control content and delivery is published elsewhere [40].

### Measures

The survey was in English and was translated into Mampruli or Nabt by the trained local enumerators at the time of interview to collect data, given the consideration that these two languages are not commonly written languages. Data collection was conducted via the CommCare platform; participants were asked to provide answers to every question before proceeding. Details for survey translation and information relevant to data collection of this program have been previously published [43].

#### Outcome

***IPV*.** Controlling behaviors, emotional violence, physical violence as well as sexual violence were assessed by the items in the Ghana Demographic and Health Survey [11]. Women were asked to indicate whether certain situations happened or not (0 = No, 1 = Yes) in relationships with their partners in the past 12 months. Example items included “He (tries/tried) to limit your contact with your family”, “Insulted or belittled you?”, and “Tried to strangle you or burn you? ”. Specifically, any acts reported under each subtype of controlling behaviors (6 items), emotional (3 items), physical (8 items)  or sexual violence (2 items) were coded as binary categories respectively for each subtype (0 = No, 1 = Yes). Physical violence and sexual violence were combined into one domain named “physical or sexual violence” in our study, given several considerations: (1) physical and sexual violence tend to be co-occurring; for example, women being physically forced to have sexual intercourse, one aspect of sexual violence [2], involves the use of physical violence; (2) Compared to other IPV subtypes, physical and sexual violence tend to lead to worse health outcomes and are often considered severe IPV as defined by the World Health Organization [2]; (3) In our data collection process, sexual and physical IPV would trigger referral mechanisms for gender-based violence services. Finally, any controlling behaviors, emotional, physical or  sexual violence were coded as having experienced any IPV in the past year (0 = No, 1 = Yes). IPV measured ~ 8 months post-intervention was the primary outcome of interest.

#### Mediators

##### Social support

The Modified Medical Outcomes Study Social Support Survey was used to measure social support (mMOS-SS) [44]. mMOS-SS has been previously used among women in Northern Ghana [45], with the authors demonstrating high internal reliability (Cronbach’s alpha = 0.90). Example of items included “If you needed it, how often is someone available to help you if you were confined to bed?” and “If you needed it, how often is someone available to love and make you feel wanted?” Women were asked to assess how often they had different types of support available from 0 (none of the time) to 4 (all of the time). Total scores had a possible range from 0 to 32. For analysis, we used the change in social support, measured by the score differences between baseline and immediate post-intervention. A higher score represented a larger increase in social support. The internal consistency measured by Cronbach’s alpha for this scale was 0.89 at baseline and 0.92 immediate post-intervention, indicating excellent internal reliability in our sample.

##### Couple communication

Couple communication was measured by the communication domain from the Couple Functionality Assessment (CFAT) [46], validated in a rural population in Malawi. The 7-item communication domain consisted of three items on constructive communication (e.g. “We express our feelings to each other”) and four items on destructive communication (e.g. “I call my husband/partner names, swear at him, or attack his character.”). Women were asked to rate their response to problems in their intimate relationships on a 5-point Likert scale from 1 (very unlikely) to 5 (very likely). The total score was calculated by adding the score for each item after the reverse coding of the four destructive items, ranging from 7 to 35. The differences between the couple communication score at baseline and immediate post-intervention were used to measure change in couple communication in the model. A higher score indicated a greater improvement in communication between couples. Cronbach’s alpha of items in this scale was 0.77 at baseline and 0.76 at immediate post-intervention, indicating acceptable internal reliability.

##### Covariates

Covariates were chosen a priori based on previous evidence on risk factors for IPV not only globally [47] but specific to African countries [48] and Ghana [17, 49]: age, education, number of pregnancies, relationship status, whether receiving sufficient support from husband, depression and IPV status at baseline. Variables including age, education, number of pregnancies, relationship status, and whether receiving sufficient support from husband were self-reported by women using a single-item question for each. Depression status and IPV status at baseline were also counted as covariates in the model. For models examing each subtype of IPV, only the same IPV subtype at baseline was used as a covariate in the corresponding model.

Socioeconomic status (SES) was assessed with the Ghana Equity Tool and two additional questions representing household wealth (i.e. a satellite dish, mobile money) recommended by the Ghana data collection team. The Ghana Equity Tool included 11 potential assets for household wealth (e.g. color television, bank account, electricity). A five-quintile wealth index based on the 13 assets was created to represent SES by the polychoric correlation principal component analysis [50, 51].

Depression was assessed with the 9-item Patient Health Questionnaire (PHQ-9), previously validated in Ghana [52]. Women were asked to evaluate how much they were bothered by listed symptoms within the past 2 weeks such as “Trouble falling or staying asleep, or sleeping too much”, and “Feeling down, depressed, or hopeless” from 0 (not at all) to 3 (nearly everyday) [53].

##### Paticipant preferences

Preferences for future iMBC/ECD programs were also assessed among participants in the intervention group immediate post-intervention, including the involvement of male partners and preferred group format (i.e male-only groups or couple-based groups). This data is reported for descriptive purposes only.

### Ethical consideration

The current study has received ethical approval from the Duke University Campus IRB (# 2019–0020) and the Navrongo Health Research Center in Ghana (# NHRCIRB314). All methods and procedures were compliant with the Helsinki Declaration (1975; revised in 2008) and followed the ethical standards of the relevant national and institutional guidelines and regulations on research involving human subjects. We obtained written informed consent forms from participants; for illiterate participants, we obtained their fingerprints and witness signatures after reading them the informed consent forms. In accordance with global best practices for research on mental health and gender-based violence and GHS recommendations, we offered a mental health referral to any participant with suicidal ideation during the survey and a domestic violence social services referral to any participant who reported physical or sexual violence in the last 12 months. Among participants who screened positive for sexual/physical IPV ~ 8 months post-intervention (*n* = 35), all were offered a social service referral for gender-based violence services but only 25.7% (*n* = 9) accepted the referral; whereas, 91% (*n* = 10) of participants who screened positive for mental health referral need (*n* = 11) accepted the referral.

### Statistical analysis

**Descriptive statistics** were used to detail the sample characteristics at baseline, mediators and outcome variables by intervention and control arms among rural women in Ghana.

#### Between-group imbalance

Given the risk of baseline imbalance in cRCTs, general linear models for continuous measures and Chi-square (or Fisher’s exact) tests for categorical measures were used to test for significant group differences (*p* < 0.10) on sample characteristic measures and identify potential covariates for the subsequent analyses other than the covariates identified a priori.

#### Sensitivity analyses

We examined any baseline variables other than those identified a priori that may be differential by missingness at immediate post intervention or ~ 8 months post intervention (*p* < 0.10). This would allow us to examine whether outcomes of interest can be assumed to be missing at random conditional on the covariates already included in the model.

#### Hypothesis testing

Two sets of hypotheses were tested for any IPV and each subtype of IPV respectively. Hypothesis 1 was examined by estimating the total effects of intervention on IPV outcomes using logistic regression models while controlling for the covariates. Hypothesis 2 was tested by estimating the indirect effects of intervention on IPV through the parallel mediation effects of social support (hypothesis 2a) and couple communication (hypothesis 2b) between intervention and IPV, wherein a multiple mediation model using the ordinary least squares regression and logistic regression models was applied. The PROCESS macro was used for testing hypothesis 2. The PROCESS macro is appropriate in the current study, allowing simultaneous estimation of the two theory-driven mediators while accounting for the covariates [54]. Model 4 (parallel multiple mediation model) from the PROCESS macro was selected for this analysis, providing the point estimates of the coefficients for each path between intervention, mediators, and outcomes as well as indirect effects through individual mediators. The 95% confidence interval (CI) of these point estimates in hypothesis 1 and hypothesis 2 was constructed using the cluster-robust standard errors by drawing 1000 bootstrap samples of the 32 clusters. For the models, two-sided statistical significance was defined by *p* < 0.05. All analyses were conducted using SAS 9.4.

#### Participant preferences

Descriptive statistics were used to summarize participant preferences.

## Results

At baseline, 374 women participated and 266 (71.12%) completed the follow-up survey ~ 8 months post-intervention. The mean age of women was 26.95 ± 6.80 years. Nearly half of the participants (48.66%) never received any formal education and 39.46% of the women were in the two lowest SES quintiles. Most of the women were married and living with their husbands (90.03%) and 44.12% of them had been pregnant four or more times (See Table 1). Enrollment and participation of women are shown in the CONSORT Diagram (See Appendix A in Supplementary information). Detailed information on the sample characteristics at baseline has been reported elsewhere [39].

**Table 1:** Sample characteristics at baseline

	Total (*n* = 374)	Control (*n* = 153)	Intervention (*n* = 221)
**Age**			
Mean (SD)	26.95 (6.80)	26.95 (6.53)	26.95 (6.99)
**Education**			
Yes	192 (51.34)	79 (51.63)	113 (51.13)
No	182 (48.66)	74 (48.37)	108 (48.87)
**SES (Asset Quintile)**			
Lowest Quintile	73 (19.73)	33 (21.57)	40 (18.43)
Second Quintile	73 (19.73)	40 (26.14)	33 (15.21)
Middle Quintile	76 (20.54)	28 (18.30)	48 (22.12)
Fourth Quintile	69 (18.65)	29 (18.95)	40 (18.43)
Highest Quintile	79 (21.35)	23 (15.03)	56 (25.81)
**Number of Pregnancies**			
One pregnancy	82 (21.93)	32 (20.92)	50 (22.62)
2–3 pregnancies	127 (33.96)	53 (34.64)	74 (33.48)
4 or more pregnancies	165 (44.11)	68 (44.44)	97 (43.89)
**Relationship Status**			
Married and living with husband	334 (90.03)	140 (91.50)	194 (88.99)
Other	37 (9.97)	13 (8.50)	24 (11.01)
**Sufficient Support from Husband**			
Yes	170 (45.82)	76 (50.33)	94 (42.73)
No	201 (54.18)	75 (49.67)	126 (57.27)
**Depression**			
Mean (SD)	6.29 (4.10)	5.54 (3.82)	6.81 (4.21)
**Self-Reported Health Status**			
Fair/Poor	58 (15.50)	18 (11.76)	40 (18.10)
Good	135 (36.10)	53 (34.64)	82 (37.10)
Very Good/Excellent	181 (48.40)	82 (53.59)	99 (44.80)
**Household Hunger (HHS)**			
Mean (SD)	0.87 (1.28)	0.82 (1.33)	0.90 (1.24)
**Social Support (MMOS)**			
Mean (SD)	21.00 (6.44)	21.20 (6.37)	20.86 (6.50)
**Couple Communication (CFAT)**			
Mean (SD)	26.92 (4.48)	27.20 (4.53)	26.72 (4.44)

*Abbreviations*: *SD* Standard Deviation; *PHQ-9* Patient Health Questionnaire 9-item, *MMOS* Modified Medical Outcomes Study Social Support Survey, *CFAT* Couple Functionality Assessment Tool, *HHS* Household Hunger Scale

The IPV prevalence was high in our sample, with 84.8% of the women experiencing some type of IPV (i.e. controlling behaviors, emotional, physical or sexual violence) in the past 12 months. Nearly 8 in 10 women (79.1%) experienced controlling behaviors. Almost half of the women (44.6%) reported emotional IPV and 38.6% reported physical or sexual IPV. The IPV prevalence was significantly higher in the intervention group compared to the control group across all subtypes of IPV as well as any IPV at baseline. We summarized IPV by intervention arm at baseline and ~ 8 months post-intervention (See Table 2).

**Table 2:** Past 12-month intimate partner violence (IPV) among women in Northern Ghana

	Baseline				~ 8 months post-intervention (19 months post-baseline)			
	n	Control (*n* = 153)	Intervention (*n* = 221)	*p*-value	n	Control (*n* = 107)	Intervention (*n* = 159)	*p*-value
**IPV: Any**	354			0.006	237			0.41
Yes	300 (84.75)	117 (78.52)	183 (89.27)		151 (63.71)	55 (60.44)	96 (65.75)	
**IPV: Controlling**	354				235			
Yes	280 (79.10)	111 (74.50)	169 (82.44)	0.07	133 (56.60)	52 (57.78)	81 (55.86)	0.77
**IPV: Emotional**	359				247			
Yes	160 (44.57)	56 (37.09)	104 (50.00)	0.02	44 (17.81)	12 (12.37)	32 (21.33)	0.07
**IPV: Physical or Sexual**	358				245			
Yes	138 (38.55)	48 (31.79)	90 (43.48)	0.02	35 (14.29)	9 (9.38)	26 (17.45)	0.08

### Between-group imbalance

SES (*p* = 0.02) and depression status (*p* = 0.003), two covariates identified a priori, were imbalanced between groups. No other sample characteristics were found to be imbalanced at baseline between intervention and control groups at the level of 0.1.

### Sensitivity analyses

No baseline variables were found to predict missingness other than the covariates identified a priori; specifically, number of pregnancies, relationship status, and IPV status at baseline predicted missingness at immediate post intervention while relationship status predicted missingness at 8 months post intervention. Missing at random conditional on the covariates included in the model was assumed for the outcome variables and complete case analysis was used without applying any imputation strategies for missing data.

### Hypothesis testing

#### Hypothesis 1

The intervention did not reduce any IPV, b = 0.04, CI = (− 0.47, 0.55), nor any subtypes of IPV based on the results of total effects (Controlling behaviors: b = − 0.09, CI = [− 0.60, 0.42]; emotional violence: b = 0.27, CI = [− 0.67, 1.21]; physical or sexual violence: b = 0.37, CI = [− 0.83, 1.56]). Thus, hypothesis 1 was not supported.

#### Hypothesis 2

According to the results on indirect effects of the intervention on any IPV through parallel mediators, the point estimate for the indirect effect from intervention through social support differences was 0.01 with a 95% bootstrap CI at cluster-level of − 0.32 to 0.34 and the indirect effect through couple communication differences was 0.02 with a 95% bootstrap CI of − 0.50 to 0.54, suggesting that the intervention’s effects on any IPV were not mediated by either of these two mediators. Similarly, the results on indirect effects also indicated that social support and couple communication were not mediating the relationships between the intervention and any subtypes of IPV (i.e. controlling behaviors, emotional, physical or sexual violence) in a statistically significant manner. Hypothesis 2a and hypothesis 2b were not supported for any IPV and for each subtype of IPV.

**Fig. 1:**
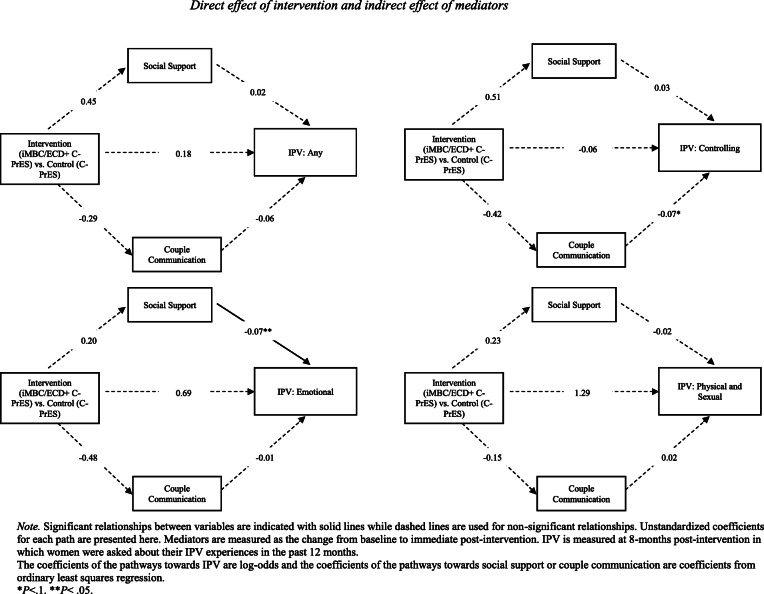
Direct effect of intervention and indirect effect of mediators

However, for a one unit increase in change in perceived social support from baseline to immediate post-intervention, there was a 7% decrease in women’s odds of experiencing emotional violence, odds ratio (OR) = 0.93, *p* = 0.007; b = − 0.07, CI = (− 0.13, − 0.02) (See Fig. 1). Similarly, per one unit increase in change in couple communication from baseline to immediate post-intervention, there was a 7% decrease in women’s odds of experiencing controlling behaviors, OR = 0.93, *p* = 0.07; b = − 0.07, CI = (− 0.14, 0.005) (See Fig. 1). Both of these effects were  from the mediators to the outcome, so such improvement cannot be attributed to the intervention.

### Participant preferences

The immediate post-intervention survey data indicated that 96% of the participants in the intervention group wanted their male partners to be involved in the iMBC intervention programs. Among those, 61% believed that their partners should be engaged in gender-specific groups (male-only groups) and 39% wanted their partners to participate together with them (couple-based groups).

## Discussion

The current study extends our empirical knowledge about the effects of mental health interventions on IPV and the underlying mechanisms by examining the impact of a group-based maternal mental health intervention on IPV reduction and the mediating roles of social support and couple communication for rural women in Ghana. Findings from this study did not support our hypotheses. The intervention, designed to improve mental health and early child development, did not reduce women’s risk for IPV compared to the control group. However, improvement in perceived social support protected women from experiencing emotional violence, though this increase was not due to the effects of intervention. In addition, better couple communication demonstrated promise in reducing controlling behaviors from their partners, though the improvement in couple communication cannot be attributed to the intervention. This study has important implications for future research and practice in IPV intervention efforts in LMICs.

There was little support for the hypothesis that the iMBC/ECD intervention would reduce IPV prevalence in the intervention group compared to the control. The results should be interpreted in light of IPV trajectories during stressful periods of pregnancy and postpartum. The trajectories of IPV during these periods remain unclear despite emerging evidence indicating a drop in IPV during the postpartum period [47]. However, it is well established that women experiencing IPV prior to or during pregnancy are more likely to continue experiencing IPV postpartum compared to those who do not [47, 55]. This is a possible reason underlying the null results of our intervention, given that a significantly higher proportion of women randomized to the intervention group experienced IPV prior to or during pregnancy and were subject to higher risk postpartum compared to women in the control group. The lack of differences in IPV prevalence reduction could also be due to the effects of the women’s groups themselves whereby all participants in both the intervention and control groups were in group-format programming. This also provided women in the control group with socialization opportunities through regular meetings, which might have helped reduce IPV. We saw similar reductions in both intervention and control groups on mental health outcomes in the main trial and similarly hypothesized that participation in a women’s group (regardless of content) could be having a greater than anticipated effect. The main trial analysis did not find a significant mental health effect, although the results favored the intervention arm. Overall, there were very low depression means in both the intervention and control groups post-intervention; 1.96, 1.40, respectively [40]. Another possible explanation is that in contexts with significant gender inequitable norms, where women have less access to resources and economic opportunities, interventions focused on the relationship-level factors might be beneficial, but not sufficient unless structural-level factors are simultaneously addressed [56], such as the shared drivers of gender inequity and poverty suggested by the syndemics theory.

The hypothesized mediating role of social support was not supported, though improvement in perceived social support was observed to reduce emotional violence. This is consistent with previous evidence that social support is a well-established factor in reducing women’s risk for IPV [26] and its negative health consequences, and thus has been the modifiable target across different IPV interventions [31]. This study was among the first to test the potential mechanisms of change for a mental health program focused on improving social support (via social activation) and reducing IPV. Again, testing this hypothesis may have been challenging given that both the intervention and control arms were group based with some naturally supportive elements via group participation. Furthermore, the interpretation of our results should be rooted in the Ghanaian context where gender inequality and poverty are prevalent (i.e. powerful drivers indicated by syndemics theory). Though the evidence generated from this study suggests increasing social support may be a viable and effective strategy in addressing IPV among women in Ghana, the social and cultural context needs to be carefully evaluated to avoid causing any unintended harm to women regarding future efforts to address IPV. Strategies with a sole focus on relationship-level factors, including social support, are not sufficient for protecting women from IPV in environments where individual risk may be high, given community norms that justify IPV and/or where there are repercussions for transgressions of rigid gender roles.

Constructive couple communication demonstrated promise in reducing controlling behaviors by male partners. The improvement in couple communication was not due to the intervention and the mediating role of couple communication was not supported. Informal feedback from participants to iMBC/ECD program staff indicated that some women felt their participation in the program improved the household dynamics and facilitated open communication with their partners about their needs, thus helping to avoid conflicts. For example, one session of the iMBC/ECD titled “Learn How to Communicate to Get Your Needs Met” focuses on communication. Couple communication could be an important factor for reducing IPV among certain subgroups. The current study extends the literature on the effectiveness of communication strategies in IPV reduction in LMICs and also moves beyond HIV-specific couple communication strategies. The results are consistent with previous evidence that couple communication was an effective strategy for IPV intervention. However, we need to interpret our results cautiously since couple communication strategies alone might be limited in their effectiveness in more severe types of IPV (e.g., physical or  sexual IPV) where women’s safety is at danger. Couple communication skills were usually used in combination with other strategies, such as problem solving skills or conflict management skills, to foster healthy intimate relationships in previous interventions effective in preventing or reducing IPV [36, 37]. The communication strategies should be strengthened and applied simultaneously with other evidence-based strategies discussed above to enhance the functionality of intimate relationships. These strategies should be further integrated into existing mental health resources to effectively address the challenges women face relevant to IPV.

Consistent with the growing research and programmatic interest to engage men in IPV interventions, most women in the intervention group indicated their interests in involving their male partners, though their preferred engagement approach differed: gender-specific groups for men or couple-based groups. With the shift from considering men as only the perpetrators of IPV towards allies in addressing IPV, interventions engaging men are promising where sustainable and effective interventions assist in transforming the inequitable gender norms [57]. The best approach to engage men has long been a topic of debate. Gender-specific groups are the standard approach in the field where women who have experienced IPV and their male partners who have perpetrated IPV are treated in separate groups in consideration of women’s safety and comfort level discussing the sensitive topic of IPV. The recommendation for couple-based approaches, however, should be made with great caution. Couple-based interventions have been controversial in the field and should only be appropriate for couples in cases of less severe IPV where women’s safety is not threatened. Couples need to be carefully evaluated to ensure that IPV is originating from situational conflicts, not driven by the desire to dominate the partner, and that women are not at risk for injury [58].

### Implications for research and practice

Future research should continue to examine the effects of mental health interventions on IPV as supported by the syndemics theory and previous empirical evidence, especially in LMICs. The knowledge will further guide us to strengthen or integrate effective components of IPV reduction such as couple communication strategies into existing mental health resources, which will more effectively promote the health of disadvantaged women faced with multiple health threats including IPV and mental health problems. This is particularly relevant in low-resource settings where IPV-specific resources are limited [59] and settings where the referrals for IPV-related services are not as acceptable as referrals to mental health resources, as in our study. Tremendous efforts have been made by the research community to reduce IPV, while the mechanisms of change underlying the effectiveness of these interventions still largely remain unexplored. Future research should elucidate mediating factors as an essential part of IPV program evaluation.

We can fill critical gaps in current scholarship by identifying the core components and mechanisms of those interventions with an impact on IPV outcomes, thus benefitting women who have experienced IPV. Future programs conducted in LMICs can further benefit participants by integrating components addressing factors beyond the relationship level. Multi-component interventions simultaneously addressing risk factors across different levels of the socio-ecological model have great promise in achieving a long-sustained impact on IPV reduction [57]. As an example, IMAGE addresses gender norms and poverty (structural-level factors) through microfinance and community mobilization to modify gender norms while providing training in individual and relationship level skills such as critical thinking and communication, leading to an over 50% reduction of IPV risk [38]. In rural Ghana, where similar structural-level factors of gender inequity and poverty are the driving force of IPV, an economic empowerment component to improve women’s autonomy and household poverty with community mobilization challenging gender norms needs to be considered in future IPV prevention programs. Engaging male partners is also a strategy accepted by women, but the format of engagement, in gender-specific groups or in couple-based groups, needs to be carefully evaluated based on the severity of the IPV to prioritize women’s safety.

### Limitations

Several limitations of this study need to be acknowledged. First, IPV over the past 12 months, the outcome of interest, was measured ~ 8 months post-intervention, thus also capturing the IPV experiences during the last four months of the intervention before the mediators were measured at immediate post-intervention. This could have led to an underestimation of our intervention effects on IPV. Another limitation is that the control arm was also delivered in group format, making it difficult to tease out the roles of social support. In addition, the measure for two mediators social support (measured by mMOS-SS) and couple communication (measured by CFAT; validated in Malawi) has not been validated in Ghana before. Also, the lack of blinding to participants, data collectors, and analysis team may introduce bias and lead to overestimation of the intervention effects. Lastly, adherence to the intervention was not controlled for in the current analysis, given that adherence was self-reported in the categories of attending none, less than half, about half, more than half, and all sessions. Although 71.6% of the women reported to have attended more than half of the intervention sessions, we acknowledge that the data available was unable to answer how the number of sessions attended was related to the IPV outcomes.

## Conclusion

Findings from the study contribute to the empirical evidence about the impact of mental health interventions on reducing IPV and the underlying mechanisms of change. Despite no observed reduction in IPV for women in the intervention group compared to those in the control group, the current study demonstrated that improvements in social support reduced women’s risk for emotional violence and increases in positive couple communication showed promise in reducing the risk of experiencing controlling behaviors by their partners, though both changes were not due to the intervention. Future research should continue to evaluate the effects of mental health interventions on IPV as well as to further illuminate the mechanisms of change to ensure the effectiveness of such interventions. Future programs in LMICs can better benefit women by addressing IPV-relevant risk factors beyond the relationship level, such as poverty and gender inequity.

## Supplementary Information

**Additional file 1.**

## Data Availability

The datasets generated and/or analyzed during the current study are not publicly available but are available via communication with the senior author (JNB) on reasonable request, with approval from Catholic Relief Services (CRS), and with IRB approval to maintain confidentiality.

## Abbreviations

IPV: Intimate partner violence
LMICs: Low- and Middle-Income Countries
PREP: Premarital Relationship Enhancement Program
IMAGE: Microfinance for AIDS and Gender Equity
iMBC: Integrated Mothers and Babies Course
ECD: Early Childhood Development
C-PrES: Community Surveillance and Targeted Education Sessions
CRS: Catholic Relief Services
GHS: Ghana Health Service
mMOS-SS: The Modified Medical Outcomes Study Social Support Survey
CFAT: Couple Functionality Assessment
SES: Socioeconomic status
PHQ-9: 9-item Patient Health Questionnaire
CI: 95% Confidence Interval
OR: Odds Ratio
